# Trust, truth and transparency: analysing the references underpinning AI-generated surgical information

**DOI:** 10.1308/rcsann.2026.0021

**Published:** 2026-03-18

**Authors:** RS Sidhu, A Selvamogan, A Boddy

**Affiliations:** ^1^ Royal Berkshire NHS Foundation Trust, UK; ^2^ University Hospitals of Leicester, UK

**Keywords:** Artificial intelligence, General surgery, Trust and transparency, Medical informatics, Bibliometrics

## Abstract

**Introduction:**

Artificial intelligence (AI) chatbots, powered by large language models, are used increasingly for disseminating surgical information, but concerns about accuracy, hallucinations and source reliability persist. This study evaluates the sources of information upon which these systems rely when producing medical information. As these models generate language without true comprehension or reasoning, assessing the credibility and nature of their referenced sources is essential to promote transparency and support evidence-based integration of AI in healthcare.

**Methods:**

Nine AI chatbots (ChatGPT-5, ChatGPT-5 Think, DeepSeek R1, DeepSeek DeepThink, Google Gemini 2.5 Flash, Grok 3, Grok 4, Perplexity Research and Perplexity Search) were queried with six standardised general surgery prompts, both with and without explicit requests for references (*n*=108 outputs); 1,249 references were extracted and assessed for quantity, authenticity, quality, source category, accessibility, geographic origin and attribution.

**Results:**

Reference provision varied: four chatbots required explicit prompting, whereas others cited consistently. Hallucination rates ranged from 0% (five models) to 34% (Grok 3). Mean quality scores differed significantly, with Perplexity Research achieving the highest score (4.08) and ChatGPT-5 the lowest (2.39), reflecting differences observed in source type. Most references originated from the US or UK. Accessibility was best in Google Gemini (100% open access, clickable citations). Explicit prompting increased reference quantity significantly in six models and quality in one.

**Conclusions:**

AI chatbots exhibit heterogeneous reference integrity, with risks of hallucinations and biases underscoring the need for prompt engineering, model refinements and ongoing evaluation. Our findings suggest ongoing caution is required in surgical contexts to ensure safe, equitable information dissemination.

## Introduction

Artificial intelligence (AI), particularly large language models (LLMs), is increasingly being integrated into healthcare across diverse settings. From supporting patient education to assisting clinical decision-making and facilitating the dissemination of medical knowledge, AI technologies offer promising opportunities to enhance the accessibility and delivery of healthcare information.^1–4^ An increasing number of people now turn to AI systems for medical advice, underscoring their expanding role as sources of health information. By rapidly generating medically orientated text and simulating expert-like dialogue, these systems have the potential to improve patient engagement and supplement evidence-based decision support.^1,3–5^

However, the widespread use of LLMs has raised important concerns about accuracy and reliability. Because LLMs generate responses by recognising and reproducing statistical patterns in text, their architecture prioritises linguistic plausibility over factual accuracy.^5,6^ Consequently, their outputs are only as reliable as the data they consume. When those data contain errors, biases or omissions, these deficiencies are reflected—and sometimes amplified—in model outputs. This has led to reports of inaccurate or misleading medical information, inappropriate clinical recommendations, and even fabricated medical advice or citations, a phenomenon known as ‘hallucination’.^7,8^ Such issues, which have already resulted in patient safety risks, disproportionately affect minority populations who are under-represented in datasets.^1,2,5^ This can lead to higher rates of misdiagnosis and inequitable care when AI systems are used for clinical decision support or information dissemination.^8,9^

Given these concerns, it is imperative to develop a clear understanding of where AI chatbots source their information when responding to medical queries. Just as clinical practice is grounded in a hierarchy of evidence that prioritises reliable and verifiable data, AI systems should likewise be evaluated on the trustworthiness of the sources they cite. Many chatbots provide lists of references following their answers, signposting the origins of their information. Although previous studies have acknowledged the presence or absence and basic validity of these references, few have examined them in detail.^3,4,10^

This study builds upon existing research by systematically evaluating the references generated by nine AI chatbots across six general surgery queries. Each reference was examined for its quantity, authenticity, quality, source category, accessibility, geographic origin and attribution to promote safer, more transparent and evidence-based integration of AI systems in healthcare.

## Methods

### Study design

This cross-sectional study evaluated the characteristics of references produced by AI chatbot-generated responses to standardised queries on common general surgical conditions and procedures. Nine AI chatbots representing a range of leading LLMs were evaluated: ChatGPT-5, ChatGPT-5 Think, DeepSeek R1, DeepSeek DeepThink, Google Gemini 2.5 Flash, Grok 3, Grok 4, Perplexity Research and Perplexity Search.

### Selection of AI chatbots and prompts

Six standardised prompts were developed to simulate typical patient enquiries related to general surgery topics (Table 1). Prompts were informed by search data from Google Trends and frequently asked questions published by reputable healthcare sources such as the Mayo Clinic and NHS England.

**Table 1:** Prompts used for AI chatbot evaluation

Prompt ID	Prompt text
1	What are the signs and symptoms of appendicitis? (Please provide references)
2	What are the risks of gallbladder removal? (Please provide references)
3	What is the recovery process like from umbilical hernia repair? (Please provide references)
4	What are the chances of recurrence after breast cancer surgery? (Please provide references)
5	What are the risks and benefits of bariatric surgery? (Please provide references)
6	What are my alternatives to surgery for sigmoid colon cancer? (Please provide references)

The six core surgical queries and their corresponding variations used to generate responses. Prompts 1–6 represent implicit requests; explicit versions of the prompts append ‘Please provide references’*.*

On 20 August 2025, each prompt was submitted to every chatbot under two distinct conditions:

1. as a standalone query;
2. with an added explicit request for citations (‘Please provide references’).

This approach allowed us to explore the effects of basic prompt engineering and confirm whether a chatbot’s failure to cite was due to incapacity or simply a lack of an explicit instruction. Before each submission, chatbot memory and conversation history were cleared.

### Data collection and evaluation

A total of 108 outputs were generated across all models. Each reference contained in these outputs was extracted and analysed individually according to seven predefined metrics (quantity, authenticity, quality, source category, accessibility, geographic origin and attribution) as detailed in Table 2.

**Table 2:** Metrics used for reference evaluation

Metric name	Metric category	Description	Coding/scale
Total number of references	Quantitative	The total number of references provided per chatbot output	Count (integer)
Publication year	Quantitative	The year the source material was published	Year (integer)
Hallucination	Binary	Indicates whether the reference is fabricated, nonexistent or irrelevant	1 = hallucinated, 0 = legitimate
Source type	Categorical	Classification of the nature of the source	Categories: Gov/Official (e.g., NICE, NHS); Academic/Nonprofit (e.g., Mayo Clinic); Professional Society/Charity (e.g., Cancer Research UK); Journal Article; Health Information Portal (e.g., WebMD); Private Practice/For-Profit (e.g., Bupa); News/Blog
Country of origin	Categorical	The country where the source originates	Country name (string)
Paywall status	Categorical	Accessibility status of the source	Categories: Open Access; Registration Required; Paywall
Source attribution in text	Ordinal	Level of in-text citation provided	5-point Likert scale: 1 = no attribution, 5 = full in-text citation
Source quality	Ordinal	Clinician-rated quality of the source	5-point Likert scale: 1 = poor, 5 = excellent

Description of the eight metrics used to assess each reference, categorised by data type. Ordinal scores for source quality and relevance were assessed by a panel of three independent clinicians*.*

The selection of evaluation metrics was guided by established frameworks for assessing the quality and credibility of online medical information. Specifically, the criteria were informed by the JAMA benchmarks, which emphasise authorship, attribution and disclosure as indicators of trustworthy health content, and the TRAAP test (Timeliness, Relevance, Authority, Accuracy and Purpose)—a validated tool for evaluating information reliability.^11–14^ Elements of the ‘Health On the Net Principles’ were also considered to ensure alignment with widely recognised standards of transparency and verifiability in digital health communication.^15^ Together, these frameworks provided a structured basis for assessing reference integrity, accessibility and evidentiary quality across AI-generated outputs.

When assessing Source Quality, a five-point Likert scale was employed. A panel of three independent clinicians (RS, AS, AB) rated these aspects, following a predefined scoring rubric discussed before assessment to ensure consistency.

### Statistical analysis

All statistical analyses were conducted using RStudio (version 2025.09.0+387). Inter-rater reliability for Source Quality scores was assessed using the intraclass correlation coefficient (ICC), demonstrating excellent agreement among the three raters (ICC=0.945, 95% confidence interval (CI): 0.938–0.951). The mean of the three ratings was then calculated to create a composite Source Quality score for each reference, which was used in subsequent inferential analyses.

## Results

### Overall performance of AI chatbots

From the 108 outputs generated by the nine AI chatbots in response to six standardised prompts under two conditions, a total of 1,249 individual references were extracted and analysed.

Table 3 presents the descriptive statistics for five reference characteristics, stratified by chatbot and prompting condition. Several models (ChatGPT-5, DeepSeek R1, DeepSeek DeepThink and Grok 3) produced no references unless explicitly prompted, whereas ChatGPT-5 Think, Grok 4 and both Perplexity models consistently supplied citations even without such instruction (Figure 1). Hallucinated references remained a persistent issue in some models, most notably Grok 3 and DeepSeek DeepThink, which exhibited high hallucination rates of 34% and 25%, respectively. When authentic references were generated, marked differences in quality and integrity emerged: ChatGPT-5 achieved the lowest mean reference quality score (2.39), whereas ChatGPT-5 Think and Perplexity Research demonstrated substantially higher scores.

**Figure 1:**
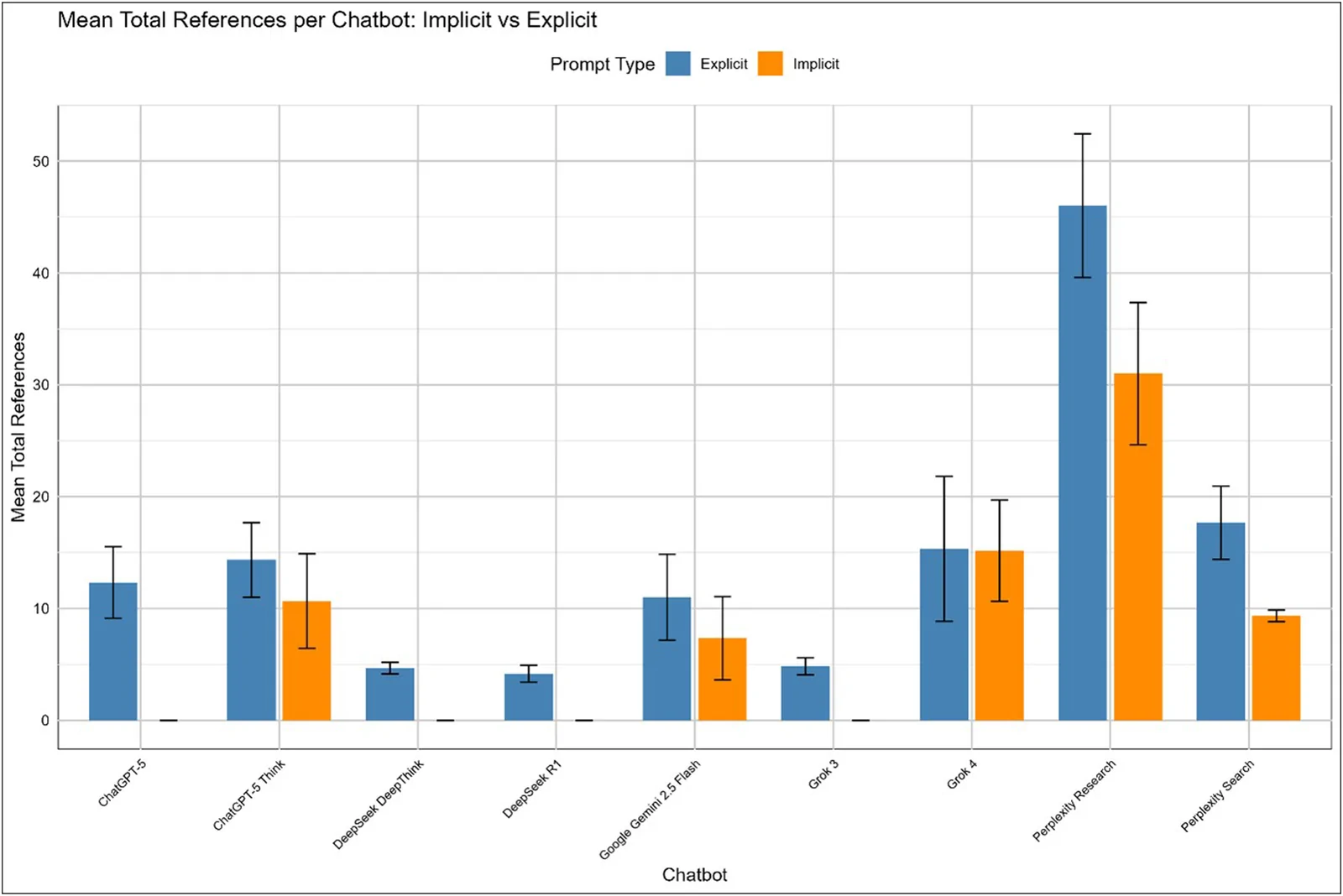
Comparison of total references provided with and without explicit prompting. The bar chart displays the total number of references generated by each chatbot, aggregated across all prompts and separated by condition: implicit requests (without a specific request for references) and explicit requests. The chart clearly illustrates that, for most models, the number of references provided increased substantially when prompted explicitly, while also highlighting the baseline performance of each model.

**Table 3:** Comparison of performance metrics by Chatbot and prompt condition

Chatbot	Prompt condition	Mean total references per output	Mean hallucination rate (%)	Mean attribution (1–5)	Mean source quality (1–5)	Open access (%)
ChatGPT-5	Explicit	12.33	1.9	4.00	2.39	98.67
ChatGPT-5	Implicit	0.00	NA	NA	NA	NA
ChatGPT-5 Think	Explicit	14.33	0.0	4.00	4.49	96.67
ChatGPT-5 Think	Implicit	10.67	0.0	4.00	4.48	100.00
DeepSeek DeepThink	Explicit	4.67	25.0	1.00	4.37	88.89
DeepSeek DeepThink	Implicit	0.00	NA	NA	NA	NA
DeepSeek R1	Explicit	4.17	10	1.00	4.35	82.61
DeepSeek R1	Implicit	0.00	NA	NA	NA	NA
Google Gemini 2.5 Flash	Explicit	11.00	0.0	5.00	3.74	100.00
Google Gemini 2.5 Flash	Implicit	7.33	0.0	5.00	3.48	100.00
Grok 3	Explicit	4.83	33.6	4.00	4.28	92.86
Grok 3	Implicit	0.00	NA	NA	NA	NA
Grok 4	Explicit	15.33	0.0	4.00	3.72	98.33
Grok 4	Implicit	15.17	0.0	4.00	3.18	98.90
Perplexity Research	Explicit	46.00	0.0	4.00	4.28	94.67
Perplexity Research	Implicit	31.00	0.0	4.00	3.78	95.35
Perplexity Search	Explicit	17.67	0.00	4.00	3.86	95.00
Perplexity Search	Implicit	9.33	0.00	4.00	3.90	98.21

The table displays mean performance metrics for each of the nine chatbots, separated by whether the prompt included an explicit request for references (‘Explicit’) or not (‘Implicit’). ‘NA’ denotes that a metric could not be calculated, typically due to the chatbot providing no references in that condition.

Figure 2 illustrates the proportional breakdown of reference source types for each chatbot, which closely reflects their quality scores. Perplexity Research cited predominantly academic journals and articles, whereas ChatGPT-5 relied heavily on health information portals and blog content. Source type distributions also varied significantly by prompt (χ²(30)=336.47, *p*<0.001), with prompts 4 and 6 (cancer-related queries) showing higher proportions of academic journals/articles and professional society/charity sources across the aggregated chatbots (Supplementary Figure A1). Figure 3 shows that the majority of references across all chatbots originated from the US or UK.

**Figure 2:**
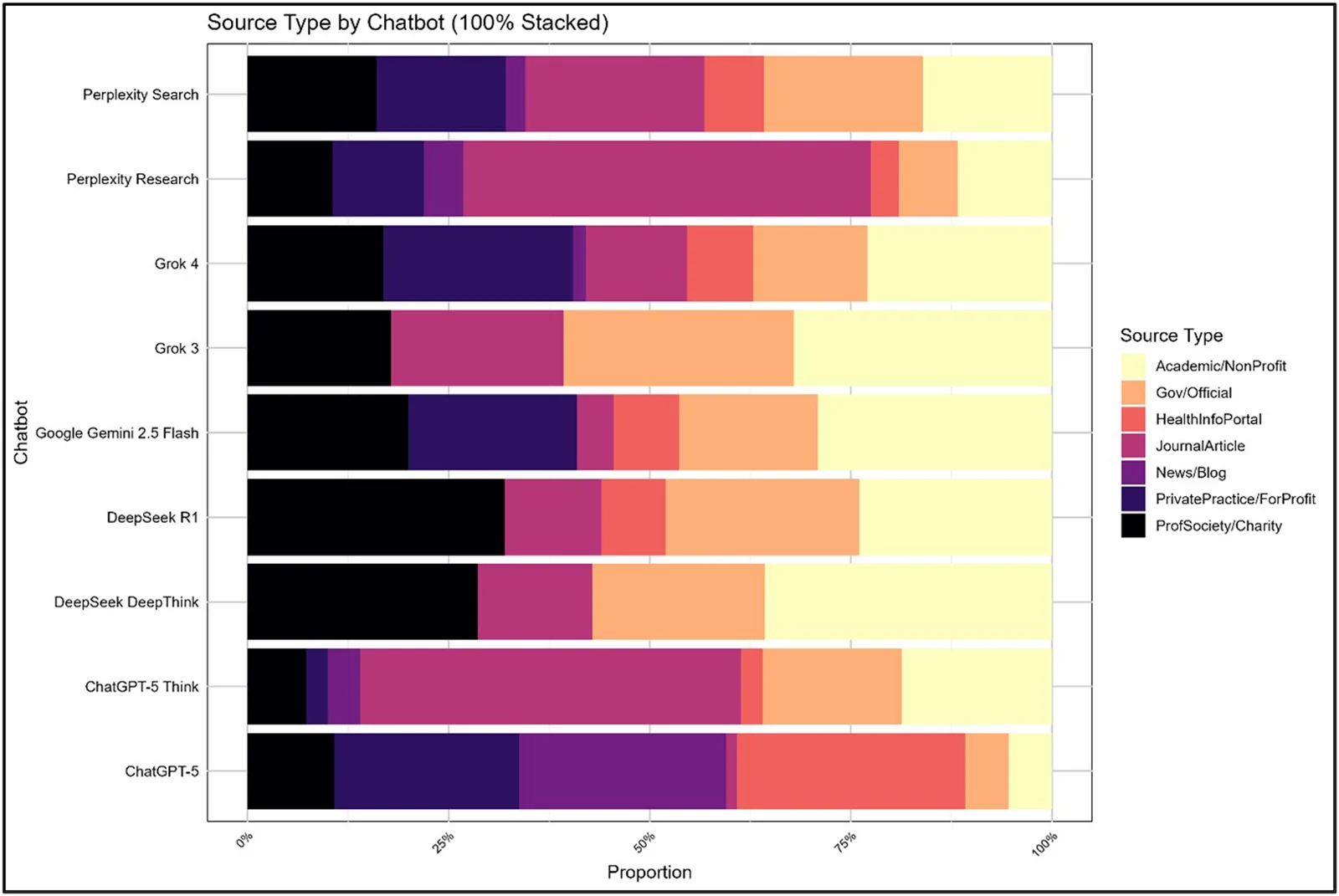
Proportional distribution of source types by AI Chatbot. A 100% stacked bar chart showing the proportional breakdown of reference source types for each AI chatbot across both prompting conditions.

**Figure 3:**
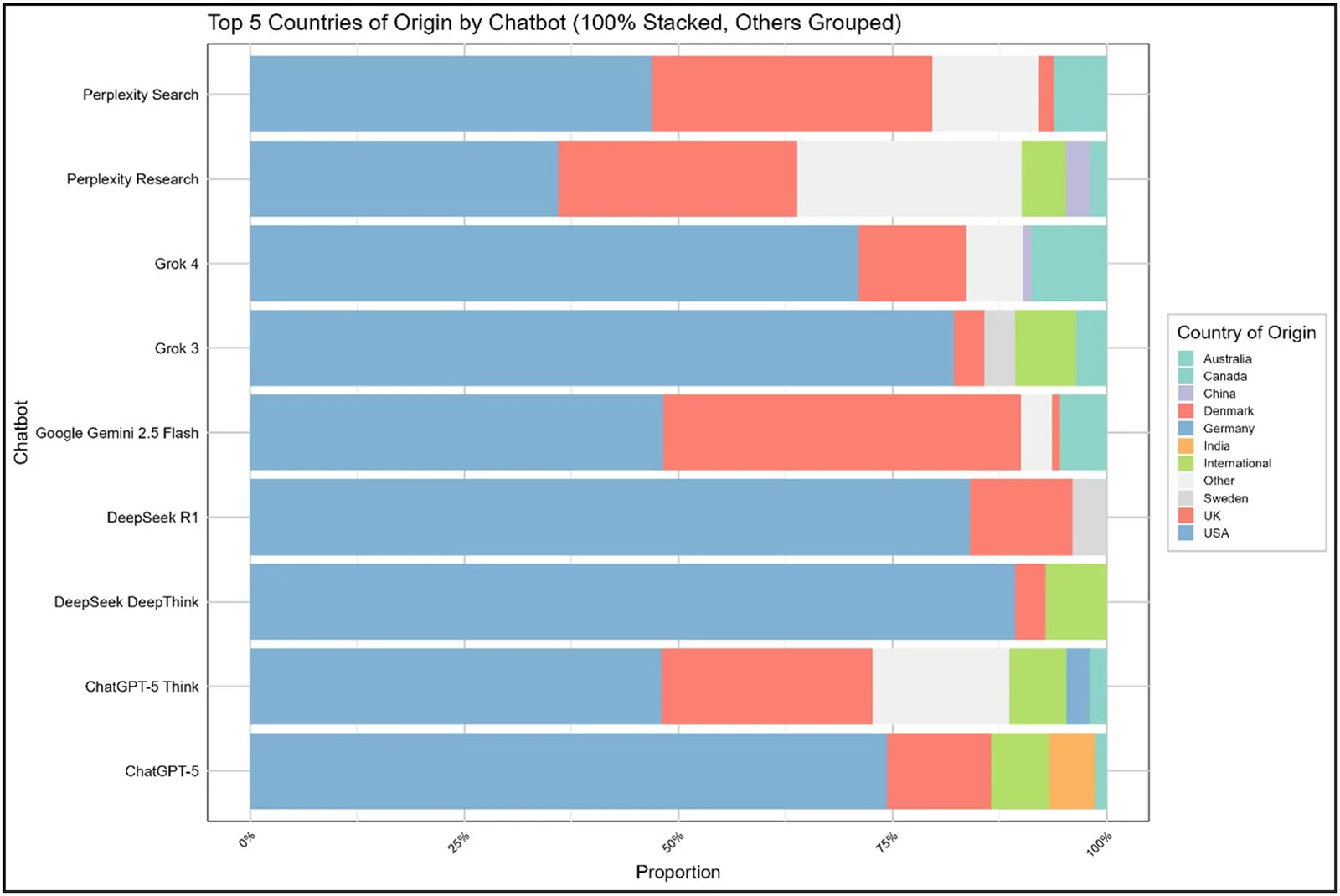
Geographical distribution of cited sources. Bar chart showing the aggregated count of references from the top five countries of origin across all nine AI chatbots.

### Statistical significance of performance differences

To assess whether the observed variations in performance were statistically significant, nonparametric tests were applied to the combined dataset. There were statistically significant differences (*p*<0.05) among the nine chatbots across all evaluated metrics, with the exception of country of origin (Table 4). Full post hoc pairwise comparisons are presented in Appendix Table A2.

**Table 4:** Summary of overall statistical significance between AI chatbots

Metric	Test used	Test statistic (χ²)	Degrees of freedom (df)	*p*-value	Statistically significant?
Total references	Kruskall–Wallis	78.56	8	<0.001	Yes
Proportion of hallucinations	Kruskall–Wallis	42.84	8	<0.001	Yes
Mean attribution score	Kruskall–Wallis	82.00	8	<0.001	Yes
Mean source quality	Kruskall–Wallis	148.82	8	<0.001	Yes
Source type	Chi-squared	445.68	48	<0.001	Yes
Country of origin	Chi-squared	407.78	432	0.647	No
Paywall status	Chi-squared	40.78	16	0.007	Yes

The table summarises results of the Kruskall–Wallis and chi-squared tests used to determine whether statistically significant differences (*p*<0.05) existed in the overall performance among the nine AI chatbots across the combined dataset.

### The impact of explicit prompting on chatbot performance

The effect of the prompt condition was evaluated by comparing each chatbot’s performance between unprompted and citation-requested outputs. Six of the nine chatbots (ChatGPT-5, DeepSeek R1, DeepSeek DeepThink, Grok 3, Perplexity Research and Perplexity Search) generated a statistically significant increase in the average number of references per response when explicitly prompted to provide citations. Perplexity Research also demonstrated a significant improvement (*p*=0.036) in average reference quality under the citation-requested condition. No other metrics showed statistically significant differences between prompt types. A full breakdown of *p*-values is provided in Appendix Table A1.

## Discussion

This study provides a comprehensive evaluation of the quality, integrity and characteristics of references generated by nine leading AI chatbots for common surgical queries. Our results reveal a high degree of performance heterogeneity, highlight the critical risks of hallucination and training data bias and underscore the complex impact of user prompting. These findings have significant implications for the safe and effective use of AI by both clinicians and patients in a surgical context.

### Reference quantity and the impact of prompting

A foundational issue identified in previous research is the inconsistent provision of citations in AI-generated medical content.^16,17^ Our descriptive findings reveal a mixed baseline of performance. Four models—ChatGPT-5 Think, Grok 4 and both Perplexity models—demonstrated an encouraging ability to consistently provide references even without specific prompting, with Perplexity Research emerging as the class leader. Google Gemini provided references in all but one of its unprompted outputs, highlighting the variability of AI responses. In contrast, four models (ChatGPT-5, DeepSeek DeepThink, DeepSeek R1 and Grok 3) produced no references when unprompted. Such inconsistency reinforces concerns that, without verifiable sourcing, AI-generated content may be perceived as less trustworthy, potentially undermining patient decision-making.^5,10^

This study demonstrates measurable progress in addressing this limitation. The simple inclusion of a reference request encouragingly led all nine chatbots to include citations in their outputs. This change of condition produced a statistically significant increase (*p*<0.05) in the average number of references for six of the nine models. These findings illustrate that current AI systems can consistently supply sources when appropriately prompted, aligning with previous evidence from Mohammad-Rahimi *et al*, who found that ChatGPT-3.5 could generate references when explicitly instructed to ‘Cite evidence where appropriate’.^18^

Notably, the in-prompt reference request also improved the quality of citations in one AI chatbot, i.e., Perplexity Research, which showed a significant rise in its mean reference quality score from 3.776 without prompting to 4.281 when prompted. Overall, these results underscore the pivotal role of prompt engineering in shaping the completeness, accuracy and evidentiary robustness of AI-generated medical content.^19^

### Reference hallucination: a persistent risk to patient safety

Although the inclusion of references marks progress towards greater transparency, ensuring that these citations are genuine and verifiable remains a critical challenge. AI models can generate confident-sounding but erroneous information, commonly referred to as hallucinations. Our study identified significant variation in the number of hallucinated references supplied across the nine AI models tested (χ²=22.162, *p*=0.0046). Encouragingly, five models (ChatGPT-5 Think, Google Gemini 2.5 Flash, Grok 4, Perplexity Research and Perplexity Search) demonstrated zero hallucinated references across all outputs, suggesting meaningful progress toward verifiable sourcing.

In stark contrast, Grok 3 exhibited the highest hallucination rate at 34%—a figure that was significantly higher than most of its peers. This pattern aligns with previous evidence that LLM outputs derive from probabilistic prediction rather than genuine reasoning.^6^ In our study, several hallucinated references mimicked high-quality sources, including fabricated articles with plausible titles and invented URLs from reputable institutions like the Mayo Clinic. Such examples are particularly concerning as they simulate the features of trustworthy evidence, making them difficult to distinguish from genuine citations.^20,21^ The risk that chatbots may fabricate or mimic authoritative sources amplifies the potential for misinformation—a concern that is especially acute in patient-facing contexts where people with lower health literacy may be more likely to accept unverified information as authoritative.^2,20–22^

### Reference quality, type and origin

Among the legitimate references produced, this study also evaluated their quality and provenance—an aspect that, to our knowledge, has not been investigated systematically in previous research on AI-generated medical text. Five chatbots achieved mean scores above four out of five: ChatGPT-5 Think (4.487), DeepSeek DeepThink (4.365), DeepSeek R1 (4.348), Grok 3 (4.281) and Perplexity Research (4.078). These high scores reflected the predominance of academically robust source types. For example, Perplexity Research and ChatGPT-5 Think demonstrated a strong scholarly orientation, with 50.7% and 47.3% of their citations classified as ‘Journal/Article’, respectively. Interestingly, variations in source distributions across prompts (Figure A1) may reflect topic-specific biases in training data; for instance, Prompt 5 (‘What are the risks and benefits of bariatric surgery?’) elicited a significantly higher proportion of health information portals compared with the cancer-focused Prompts 4 and 6, potentially due to a higher proportion of commercialised content in bariatric-related online resources.

ChatGPT-5 recorded the lowest mean quality score (2.394), and post hoc analysis confirmed this was significantly lower than every other model. Its poor performance was attributable largely to a reliance on nonacademic sources, with 54.0% of references originating from Health Information Portals (28.38%) and News/Blog content (25.68%). A closer examination revealed that ChatGPT-5 frequently cited Wikipedia, appearing 11 times in six outputs.

This pattern raises important concerns about the reliance of some AI models on low-quality or nonpeer-reviewed sources. For example, although widely accessible, multiple studies have demonstrated that Wikipedia’s medical information is often less accurate and less comprehensive than established clinical references, with errors of omission being common.^23–26^ Furthermore, its open-editing model permits continual modification and potential vandalism, especially on contentious or emerging topics, which further undermines its reliability as a medical source.^27^ These findings highlight the risk that AI systems trained or reinforced on such material may inadvertently propagate inaccuracies or misinformation, emphasising the need for greater transparency and quality control in their reference generation.

Although no statistically significant differences were observed in the geographical distribution of references across chatbots, most citations originated from the US or the UK, indicating that the LLMs draw primarily from an Anglo-American information ecosystem. This pattern aligns with the global concentration of medical research, where approximately 83% of clinical trial sites are located in OECD countries, predominantly the US, whereas only 5% are situated in lower-middle or low-income regions.^28^ The under-representation of global populations in clinical research directly limits the generalisability of findings, thereby increasing the risk of inequitable care and adverse outcomes.^29–35^ As AI systems become more accessible and widely adopted worldwide, particular attention must be paid to these underlying geographic and demographic biases embedded in their training data. Unlike human clinicians, who can account for a patient’s cultural, socioeconomic and regional context, AI models trained primarily on Western-centric sources may perpetuate structural inequities in healthcare information and advice, posing a risk of differential or inappropriate guidance for under-represented populations.

### Comparison of advanced versus standard chatbot versions

The significant difference in reference quality between ChatGPT-5 and ChatGPT-5 Think highlights how variations in model architecture and processing methods can influence directly the reliability of AI-generated medical information. Although both systems are built upon the same underlying LLM, the ‘Think’ variant integrates enhanced reasoning and retrieval mechanisms that appear to improve citation accuracy and source integrity. The higher mean source quality observed for ChatGPT-5 Think suggests that such refinements may enable more discerning selection of credible references and reduce reliance on low-quality or nonacademic material. Similar patterns were observed in other model comparisons in this study. Grok 4 markedly outperformed Grok 3 in both reference quantity and accuracy, demonstrating an apparent elimination of hallucinated citations. These findings indicate that more advanced versions of AI models may hold greater potential for producing accurate, higher-quality medical information, although continued evaluation of their reference sourcing remains essential.

### Attribution and accessibility

A critical component of a usable reference is its attribution quality—the ease with which a user can verify the source in the body of the AI’s answer. This metric again showed a statistically significant difference across models (χ²=82.00, *p*=1.93×10^−14^). The class leader, Google Gemini 2.5 Flash, achieved the maximum possible rank owing to its superior accessibility features^5^: outputs included clickable in-text citations that, when hovered over, highlighted the relevant text passage and, when clicked, directed the user to the corresponding section of the external webpage. This granular level of linkage dramatically enhances both accessibility and verifiability. Importantly, Google Gemini was also the only chatbot in the cohort to provide 100% open-access references, meaning that all cited sources were freely available to users without paywall restrictions. Such open accessibility represents a crucial dimension of reference quality, as it ensures equitable access to evidence and facilitates independent verification of claims. These design and accessibility features are particularly important given the known prevalence of hallucinated or low-quality references in AI-generated text; by allowing users to trace each claim directly to its source, transparent attribution serves as a safeguard against the uncritical acceptance of fabricated or unreliable information.

In sharp contrast, models such as DeepSeek DeepThink and DeepSeek R1 consistently received the lowest possible rank. These systems typically produced only a static list of references appended to the end of the output, often lacking functional or clickable links and offering no mechanism to determine which specific statement was supported by which source. Moreover, 7.41% and 12.50% of their cited sources, respectively, led to content behind paywalls, further limiting accessibility. This deficiency substantially restricts the user’s ability to critically appraise the information presented and undermines the very purpose of providing citations in the first place.

### Limitations

This study has several limitations that should be acknowledged. First, the analysis was restricted to nine AI chatbots and a limited set of standardised general surgical prompts, which may not fully capture performance across other medical disciplines or question types. Second, although the evaluation framework incorporated validated criteria and clinician scoring to enhance objectivity, the assessment of reference quality and relevance inevitably involved an element of subjectivity. Additionally, as the geographic and linguistic scope of the prompts was limited to English-language inputs, the findings may not generalise to non-English or culturally localised contexts.^8,9^ Finally, the rapid evolution of AI models means these results may become outdated, as newer versions could outperform those evaluated here. Given this rapid development, continuous monitoring of AI-generated healthcare information is critical to maintain quality, accuracy and safety, promptly identifying emerging risks.^36^

## Conclusions

This study provides one of the first detailed evaluations of reference generation across multiple AI chatbots in response to standardised surgical queries. The findings reveal substantial variability in the quantity, quality and verifiability of citations, with significant differences observed between models and prompting conditions.

Perplexity Research consistently performed well across all evaluated metrics, producing references in every output, achieving a high average number of citations (38.5) per response, and demonstrating excellent source quality with no hallucinated references. These results suggest it may currently represent one of the more reliable sources of AI-generated medical information. However, its accessibility remains limited, as unrestricted use requires a paid subscription.

Among more widely accessible chatbots, considerable concerns persist. High rates of hallucinated references and reliance on lower-quality sources risk the dissemination of inaccurate or misleading medical information, issues that warrant particular caution in clinical or patient-facing contexts. Universal challenges, such as the predominance of Anglo-American references, further reflect broader global imbalances in medical research and underscore the need for greater representativeness and inclusivity in AI training datasets.^9^

Overall, these findings highlight both the promise and the limitations of contemporary AI systems in delivering trustworthy medical information. Ensuring that such technologies contribute safely and equitably to healthcare will require ongoing evaluation of data provenance, citation integrity and transparency mechanisms. AI tools should be regarded as adjuncts to, rather than replacements for, professional clinical communication. Given the rapid evolution of LLMs, continued interdisciplinary research will be essential to guide the responsible integration of these systems into clinical education, decision support and patient engagement.

## Competing interests

The author/s declare no competing interests.

## Funding

The author/s received no financial support for the research, authorship and/or publication of this article.

## Ethics approval and consent to participate

Not applicable.

## Author contributions

**R Sidhu:** Conceptualization, Data curation, Formal analysis, Investigation, Methodology, Project administration, Resources, Validation, Visualization, Writing – original draft, Writing – review & editing. **A Selvamogan:** Conceptualization, Data curation, Formal analysis, Investigation, Methodology, Project administration, Resources, Validation, Visualization, Writing – original draft, Writing – review & editing. **A Boddy:** Conceptualization, Data curation, Investigation, Methodology, Supervision, Writing - review & editing.

## Artificial Intelligence

The author/s declare that no AI was used to conduct the study or prepare the manuscript.

## Supplementary Information

The online version contains supplementary material available at https://doi.org/10.1308/rcsann.2026.0021
